# Low temperature synergistically promotes wounding-induced indole accumulation by INDUCER OF CBF EXPRESSION-mediated alterations of jasmonic acid signaling in *Camellia sinensis*

**DOI:** 10.1093/jxb/erz570

**Published:** 2020-01-04

**Authors:** Ying Zhou, Lanting Zeng, Xingliang Hou, Yinyin Liao, Ziyin Yang

**Affiliations:** 1 Key Laboratory of South China Agricultural Plant Molecular Analysis and Genetic Improvement and Guangdong Provincial Key Laboratory of Applied Botany, South China Botanical Garden, Chinese Academy of Sciences, Tianhe District, Guangzhou, China; 2 Center of Economic Botany, Core Botanical Gardens, Chinese Academy of Sciences, Tianhe District, Guangzhou, China; 3 The James Hutton Institute, UK

**Keywords:** Aroma, *Camella sinensis*, indole, jasmonic acid, multiple stress, tea, volatile

## Abstract

Plants have to cope with various environmental stress factors which significantly impact plant physiology and secondary metabolism. Individual stresses, such as low temperature, are known to activate plant volatile compounds as a defense. However, less is known about the effect of multiple stresses on plant volatile formation. Here, the effect of dual stresses (wounding and low temperature) on volatile compounds in tea (*Camellia sinensis*) plants and the underlying signalling mechanisms were investigated. Indole, an insect resistance volatile, was maintained at a higher content and for a longer time under dual stresses compared with wounding alone. CsMYC2a, a jasmonate (JA)-responsive transcription factor, was the major regulator of *CsTSB2*, a gene encoding a tryptophan synthase β-subunit essential for indole synthesis. During the recovery phase after tea wounding, low temperature helped to maintain a higher JA level. Further study showed that CsICE2 interacted directly with CsJAZ2 to relieve inhibition of CsMYC2a, thereby promoting JA biosynthesis and downstream expression of the responsive gene *CsTSB2* ultimately enhancing indole biosynthesis. These findings shed light on the role of low temperature in promoting plant damage responses and advance knowledge of the molecular mechanisms by which multiple stresses coordinately regulate plant responses to the biotic and abiotic environment.

## Teaser

INDUCER OF CBF EXPRESSION interacts with JASMONATE ZIM-DOMAIN proteins to relieve repression of MYC2 in tea plants, leading to enhanced accumulation of the insect resistance-associated volatile indole.

## Introduction

Environmental stress factors, such as temperature changes, drought, salinity, and herbivore attack, generally affect plant growth. To survive, plants must cope with these stress factors individually or, more commonly, in combination. Defense volatiles, including terpenoids and green-leaf volatiles, are commonly induced in plants by different abiotic and biotic stresses, including salinity (Lee and Seo, 2014), chilling (Cofer *et al.*, 2018*a*), UV-B light (Mauro *et al.*, 2014; Maja *et al.*, 2016), and herbivore attack (Baldwin *et al.*, 2002; Allmann and Baldwin, 2010), and play important roles in resisting these stresses. Among induced volatiles, indole, a derivative of the shikimate pathway, has recently emerged as an insect resistance volatile in plants (Erb *et al.*, 2015; Ye *et al.*, 2018). Studies have revealed that indole mounts a primary insect defense as a caterpillar toxin (Veyrat *et al.*, 2015), while acting as a priming signal between neighboring plants that prepares them for insect attack (Erb *et al.*, 2015). In addition, indole has also been reported to play a role in attracting parasitic wasps in the presence of caterpillars (D’Alessandro *et al.*, 2006; Ye *et al.*, 2018). These findings support the multifaceted function of indole in tritrophic interactions of plants, herbivores, and their natural enemies.

Jasmonic acid (JA) is a well-known phytohormone that regulates plant defense against both biotic and abiotic stresses (Howe *et al.*, 2018). Previous studies have demonstrated that it functions throughout the plant kingdom as an elicitor of secondary metabolite production and is rapidly synthesized and initiates defense response upon mechanical damage or insect attack (Zhou and Memelink, 2016). Furthermore, it has been reported that JA enhances the cold tolerance of plants by positively regulating the INDUCER OF CBF EXPRESSION (ICE)–core binding factor (CBF) pathway (Hu *et al.*, 2013; Sharma and Laxmi, 2015). In addition, its interaction with other phytohormones, such as ethylene, abscisic acid (ABA), and gibberellic acid (GA), helps to maintain the balance of plant growth and defense (Hu *et al.*, 2017).

*De novo* synthesis of JA starts from α-linolenic acid (Wasternack, 2007), with coronatine insensitive 1 (COI1)/JAZs/MYC2 as the core JA signaling module (Chini *et al.*, 2009). The F-box protein COI1 plays a central role in the JA signaling pathway and is essential for JA-dependent responses (Feys *et al.*, 1994; Xie *et al.*, 1998). MYC2, a basic helix–loop–helix (bHLH) family transcription factor, is the best known positive regulator of JA-dependent responses (Lorenzo *et al.*, 2004). Jasmonate ZIM-domain (JAZ) protein acts as the molecular connection between COI1 and MYC2 (Thines *et al.*, 2007). Under stress-free conditions, JAZ directly binds to MYC2, which represses MYC2 activity and subsequent JA-responsive gene expression. Jasmonoyl-isoleucine (JA-Ile) accumulation, induced by environmental stresses, triggers COI-dependent degradation of JAZ, thus releasing MYC2 to activate a downstream JA response. To balance defense and growth, multiple negative feedback loops, such as JAZ protein *de novo* synthesis, attenuate the JA response and maintain homeostasis of endogenous JA (Chini *et al.*, 2007; Chung *et al.*, 2008).

Tea [*Camellia sinensis* (L.) O. Kuntze] is an economically important perennial evergreen woody tree species that is used to prepare the second most popular global beverage after water (Tounekti *et al.*, 2012). A large number of secondary metabolites are produced in tea, especially volatiles. These volatiles play important roles in mediating physiological processes, such as pollination, natural enemy attraction, and herbivore repulsion, and quickly change in response to biotic and abiotic stresses, such as herbivore attack and unsuitable temperatures (Kessler and Baldwin, 2001; Copolovici *et al.*, 2012). Therefore, tea is an ideal model for investigating volatile-mediated stress response. Owing to the tea plant’s poor tolerance to lower temperatures, sudden frost in winter or early spring can cause significant losses in tea production (Li *et al.*, 2018). Herbivore attack, which commonly causes wounding, is the major biotic stress encountered by tea trees in the natural environment. In South China, the average winter temperature is ~10–15 °C (from December to February). Many pests in tea plantations have been shown to maintain activity at such temperatures. According to our records on the tea green leafhopper [*Empoasca* (*Matsumurasca*) *onukii* Matsuda], kept from 2010 to 2018, ~10% of the peak population of this insect can be found during winter. Another major tea pest, *Thrips hawaiiensis*, has been shown to maintain development and reproduction at 10–15 °C (Murai, 2001). Understanding the effect of combined stresses and the molecular mechanism underlying stress response will provide opportunities for engineering tea plants and/or improving farming practices to increase tea production.

In this study, we investigated the interactive effects of wounding and subsequent low temperature on plant volatile metabolites. Compared with mechanical wounding alone, the dual stresses enhanced the biosynthesis of the anti-insect-related volatile indole. Our investigation also found that low temperature maintained higher JA content, which was triggered by wounding, for a longer time. Further, we showed that indole levels were regulated by CsMYC2a and JA signaling. Competitive binding of CsICE2, a core regulator in the low temperature response, to CsJAZ2 may play a role in releasing CsMYC2a and further promoting indole biosynthesis under dual stresses.

## Materials and methods

### Plants and treatments

Tea (*C. sinensis* var. Jinxuan) shoots were obtained in September from the Tea Research Institute, Guangdong Academy of Agricultural Sciences (Yingde, China). The tea field was covered with gauze to prevent insect attack. In order to remove the jasmonate effect caused by picking, picked tea shoots were incubated at 25 °C for >24 h before further study. For wounding, one bud and three leaves were pierced with a needle. Each leaf was pierced 10 times and each bud was pierced twice. Wounded and unwounded tea shoots were incubated at specific temperatures under light/dark cycles of 16 h/8 h in a plant incubator. Each treatment was performed in three replicates. One bud and three leaves were collected at 0, 1, 4, 8, 16, and 32 h. The collected samples were immediately frozen in liquid N_2_ and stored at –80 °C until use.

For JA treatment, tea shoots were incubated for 10 h in 2.5 mM JA dissolved in 0.5% ethanol, as described previously (Zeng *et al.*, 2017). Tea shoots incubated in 0.5% ethanol were used as a control. Each treatment was performed in three replicates. One bud and three leaves were collected, immediately frozen in liquid N_2_, and stored at –80 °C prior to use.

The leaves of 4-week Arabidopsis Col-0 plants (light/dark, 16/8 h) were wounded with needles 10 times per leaf. The wounded and non-wounded plants were incubated at 4, 15, 22, and 30 °C with light/dark cycles of 16/8 h in a plant incubator. Leaves of five plants comprised one replicate. The leaves were harvested after 0, 1, 4, 8, 16, and 32 h and immediately frozen with liquid N_2_. Samples were stored at –80 °C until used.

### Phytohormone analysis

Finely powdered tea leaves (300 mg) were extracted with ethyl acetate (3 ml). d_6_-ABA (50 ng), d_4_-salicylic acid (d_4_-SA) (50 ng), and d_5_-JA (25 ng) were added as internal standards. d_5_-JA was used as an internal standard for both JA and JA-Ile (Pan *et al.*, 2010). The mixture was vortexed for 30 s and then sonicated on ice for 20 min. The extractant was then centrifuged at 12 000 *g* for 5 min at 4 °C. The upper phase (2.5 ml) was transferred to a new tube and the solvent was evaporated under N_2_ flow. The resulting pellet was redissolved in methanol (200 µl).

Phytohormones were analyzed using ultra-performance liquid chromatography/quadrupole time-of-flight mass spectrometry (UPLC-QTOF-MS) on an Acquity UPLC I-Class/Xevo G2-XS QTOF instrument (Waters Corporation, Milford, MA, USA) equipped with an AQUITY UPLC BEH C18 column (Waters Corporation, 2.1 mm×100 mm×1.7 µm). For the analysis of JA, ABA, and SA, distilled water containing 0.1% (v/v) formic acid (A) and methanol containing 0.1% (v/v) formic acid (B) were used as the mobile phase. The elution gradient was initiated with 30% B for 4 min, and then increased linearly to 65% B over 15 min. For the analysis of JA-Ile, distilled water containing 0.1% (v/v) formic acid (A) and acetonitrile containing 0.1% (v/v) formic acid (B) were used as the mobile phase. The elution gradient was initiated with 35% B, and then increased linearly to 50% B over 10 min. The flow rate was 0.25 ml min^–1^ and the column temperature was 40 °C. The MS conditions were as follows: capillary voltage, 2.5 kV; source temperature, 100 °C; desolvation temperature, 350 °C; cone gas flow, 50 l h^–1^; and desolvation gas flow, 600 l h^–1^.

### Subcellular localization

The ORFs of *CsTSA*, *CsTSB1*, *CsTSB2*, and *CsTSB3* were cloned into PSAT6-EYFPN1. The constructed plasmid was transformed into Arabidopsis mesophyll protoplasts as described by Yoo *et al.* (2007). Briefly, the lower epidermis of leaves was removed using tape as described previously (Wu *et al.*, 2009). The leaf residue was enzymolyzed in enzyme solution at room temperature for 3 h to obtain mesophyll protoplasts. Plasmids (10 µl, 1 µg µl^–1^) were transformed into protoplasts using polyethylene glycol (PEG) 4000. Protoplasts were incubated for 12 h at 22 °C after transformation. The fluorescence of yellow fluoprescent protein (YFP) was observed using confocal microscopy (Zeiss LSM 510, Carl Zeiss, Jena, Germany).

### RNA extraction, cDNA library preparation, and transcriptome sequencing

Total RNA was extracted from tea leaves (1 g) using the cetyltrimethylammonium bromide (CTAB) method. RNA integrity was confirmed using the Agilent 2100 Bioanalyzer with a minimum integrity number of 8. mRNA was purified from total RNA using oligo(dT) magnetic beads and then fragmented. The cDNA library was created using these cleaved RNA fragments and sequenced using the Illumina Hiseq 2000 platform. To estimate expression levels, data were expressed as reads per kilobase per million mapped reads by calculating the mapped reads (total exon reads/total mapped reads in millions×exon length in kb) for each gene.

### Yeast two-hybrid assay

Full-length ORFs encoding CsJAZs and CsICEs were amplified by PCR using PrimeSTAR DNA polymerase (Takara, Dalian, China). The ORFs of JAZs and ICEs were cloned into pGBKT7 and pGADT7, respectively. Y2HGold yeast cells were co-transformed with specific bait and prey constructs using the lithium acetate (LiAC)/PEG method (Gietz *et al.*, 1995). The transformed yeast cells were grown on SD/-Trp/-Leu/-His/-Ade medium for the interaction test. Transformed yeast cells plated on SD/-Trp/-Leu were used as positive controls. Empty pGBKT7 and pGADT7 vectors were used as negative controls.

### Bimolecular fluorescence complementation (BiFC) assay

The ORFs of CsJAZ2 and CsICEs were cloned into the pGreen binary vector HY105 containing C- or N-terminal fusions of enhanced YFP (EYFP) to generate *35S*:*JAZ2-EYFP*^*C*^ and *35:ICEs-EYFP*^*N*^. Arabidopsis mesophyll protoplasts were prepared as described previously (Wu *et al.*, 2009). Each constructed vector (5 µg, 1 µg µl^–1^) was co-transformed into Arabidopsis protoplasts as described previously (Yoo *et al.*, 2007), which were then incubated for 12 h at 22 °C. The fluorescence of the YFP was observed using confocal microscopy (Zeiss LSM 510, Carl Zeiss).

### Transient expression assay

The 1854 bp *CsTSB2* promoter (*pCsTSB2*) was cloned using a genome walking kit (Clontech) and confirmed by sequencing. Mutation of the *CsTSB2* promoter *mpCsTSB2* to create three mutated G-boxes (CACGTG to CGATGG) was achieved by gene synthesis. The *CsTSB2* promoter was cloned into HY107 containing the β-glucuronidase (GUS) gene to construct *pCsTSB2:GUS* and *mpCsTSB2:GUS* vectors. The ORFs of CsMYC2s, CsJAZ2, and CsICEs were cloned into pGreen-35S to construct *35S:CsMYC2s*, *35S:CsJAZ2*, and *35S:CsICEs* effector constructs. Construct *35S:LUC* was used as an internal control to evaluate protoplast transformation efficiency. Arabidopsis mesophyll protoplasts were prepared as described by Wu *et al.* (2009) and transformed as described as Yoo *et al.* (2007). GUS activity was assayed as described by Yoo *et al.* (2007). Luciferase activity was assayed using a Luciferase Assay System (Promega, Madison, WI, USA). The relative GUS activity was normalized to the luciferase activity.

### Electrophoretic mobility shift assay

A portion of CsMYC2a cDNA (corresponding to amino acid residues 401–668) was cloned into pET32a, and the construct was transformed into *Escherichia coli* Rosetta. Isopropyl-β-d-thiogalactoside (IPTG; 0.1 mM) was added to induce the expression of His-tagged recombinant CsMYC2aΔN protein at 20 °C for 16 h. Recombinant CsMYC2aΔN protein was purified with Ni-Sepharose (GE Healthcare, Chicago, IL, USA). EMSA was performed using the LightShift Chemiluminescent EMSA Kit (ThermoFisher Scientific, Waltham, MA, USA). Binding buffer contained 2.5% glycerol, 50 mM KCl, 5 mM MgCl_2_, and 10 mM EDTA. Binding reactions were incubated at room temperature for 20 min.

### Indole analysis

To assay internal indole, finely powdered tea leaves (200 mg) were extracted for 6 h with CH_2_Cl_2_ (700 µl) containing d_7_-labeled indole as an internal standard. The extracts were dried over anhydrous sodium sulfate. Extracts (1 µl) were subjected to GC-MS analysis. To assay the emission of indole, 10 tea shoots containing one bud and three leaves were placed in a beaker sealed with aluminum foil. Solid-phase microextraction (SPME; divinylbenzene/carboxen/polydimethylsiloxane, Supelco Inc.) was used to collect emission volatiles for 20 min at 25 °C, which were then subjected to GC-MS (GCMS-QP2010 SE, Shimadzu, Kyoto, Japan) analysis using a SUPELCOWAX 10 column (30 m×0.25 mm×0.25 µm, GL Sciences, Tokyo, Japan). The GC-MS temperature program was as follows: initial temperature, 60 °C for 3 min; increased to 240 °C at 4 °C min^–1^; and maintained at 240 °C for 20 min. Full scan mode (*m*/*z* 40–200) was used.

### Quantitative real-time PCR (qRT-PCR) analysis

Total RNA was isolated from finely powdered tea leaves (100 mg) using the Quick RNA Isolation Kit (Huayueyang, Beijing, China). First-strand cDNA was synthesized from total RNA (500 ng). The qRT-PCR system contained iTaq Universal SYBR Green Supermix (2×) (5 µl; Bio-Rad, Hercules, CA, USA) and 0.4 µM of each specific forward and reverse primer. The primers used are listed in Supplementary Table S1 at *JXB* online. Elongation factor 1-α (*CsEF* genes) was used as an internal reference. qRT-PCR was conducted on a Roche LightCycler 480 system (Roche Applied Science, Mannheim, Germany) under the following conditions: one cycle of 95 °C for 30 s, 40 cycles of 95 °C for 5 s, and 60 °C for 30 s. A melt curve was performed at the end of each reaction to verify PCR product specificity. The 2^−Δct^ method was used to calculate relative expression levels (Livak and Schmittgen, 2001).

### Statistical analysis

Statistical analysis, including Student’s *t*-test and one-way ANOVA followed by Duncan’s multiple test, was performed using SPSS software.

### Gene accession numbers

The sequences reported herein have been deposited in the GenBank database. The accession numbers of these genes are: MK336376 (*CsJAZ1*), MK336377 (*CsJAZ2*), MK336373 (*CsJAZ3*), MK336374 (*CsJAZ4*), MK336375 (*CsJAZ5*), MK336379 (*CsJAZ6*), MK336380 (*CsJAZ7*), MK336378 (*CsJAZ8*), MK336381 (*CsICE1*), MK336382 (*CsICE2*), MK336383 (*CsMYC2a*), MK336384 (*CsMYC2b*), MK336385 (*CsMYC2c*), MG708228 (*CsLOX*), AHY03308 (*CsAOS*), AHY03309 (*CsAOC*), LOC114260773 (*CsJAR*), KX022968 (*CsTSA*), KX022969 (*CsTSB1*), KX022970 (*CsTSB2*), and KX022971 (*CsTSB3*).

## Results

### Effect of dual stress treatment on indole accumulation in tea

**Fig. 1.**
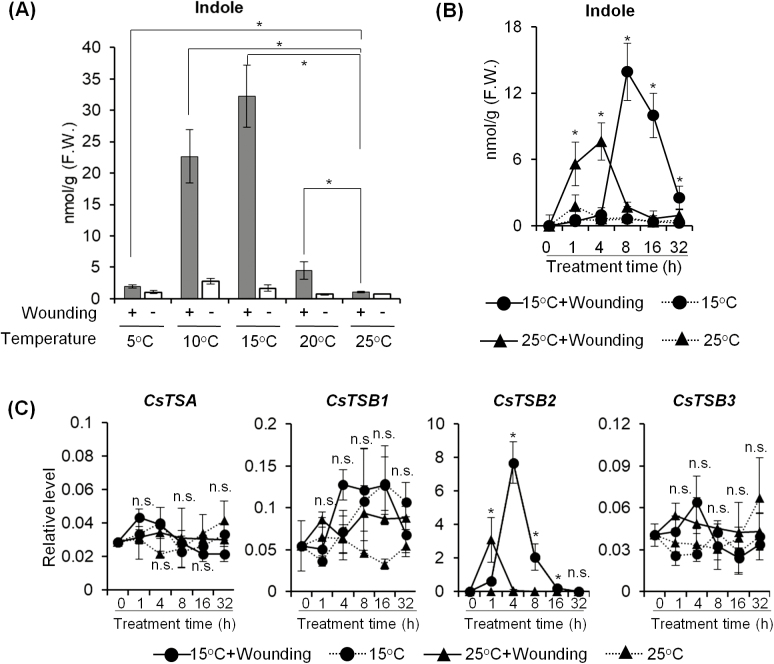
Accumulation of indole induced by increased *CsTSB2* expression level under dual stresses. (A) Effect of low temperature on indole formation 16 h after mechanical wounding. The leaves of tea shoots were wounded with a syringe needle. The tea shoots were then incubated at different temperatures (5, 10, 15, 20, and 25 °C) after mechanical wounding. Tea shoots incubated at 25 °C served as the control. Tea leaves were collected after incubating for 16 h. Internal indole was extracted with CH_2_Cl_2_ and isotope-labeled indole was added as an internal standard. The indole content of the tea leaves was analyzed by GC-MS. Asterisks (*) indicate statistically significant differences (Student’s *t*-test; *P*≤0.05). All data are means of three independent experiments, and error bars represent ±SD. (B) Time course of internal indole analysis of *C. sinensis* leaves under different treatments. Internal indole was extracted with CH_2_Cl_2_ and isotope-labeled indole was added as an internal standard. Indole was analyzed by GC-MS. * indicates statistically significant differences between wounded plants incubated at 15 °C and 25 °C (Student’s *t*-test; *P*≤0.05). All data are means of three independent experiments, and error bars represent ±SD. (C) Gene expression level of *CsTSB2*, showing more significant up-regulation under dual stresses. The gene expression level was analyzed by qRT-PCR and calculated using the 2^−Δct^ method. * indicates statistically significant differences between wounded plants incubated at 15 °C and 25 °C (Student’s *t*-test; *P*≤0.05; n.s.=not significant). All data are means of three independent experiments, and error bars represent ±SD.

Plant volatiles are considered to be defense metabolites, and their abundance changes under environmental stress (Howe and Jander, 2008; Vickers *et al.*, 2009). Studies showed that wounding induces the accumulation of various volatiles, such as linalool, (*Z*)-3-hexen-1-ol, and indole (Frey *et al.*, 2000; Mithöfer *et al.*, 2005; Zhang *et al.*, 2016). Chilling (at 0–15 °C) is an environmental stress that can cause serious physiological dysfunction and limit plant growth (Prasad *et al.*, 1994). However, how plant volatiles change under exposure to both wounding and low temperature stresses remains unknown. To analyze the impact of the dual stresses on volatile metabolites, tea shoots were incubated at different temperatures (5, 10, 15, 20, and 25 °C) for 16 h after mechanical wounding. The results showed that mechanical wounding induced anti-insect-related volatile indole. Interestingly, indole was significantly higher in wounded tea leaves incubated at lower temperatures than in those incubated at 25 °C (Fig. 1A). Wounded tea leaves incubated at 15 °C contained the highest indole content. To further investigate the changes in indole content under dual stresses over time, tea shoots with or without mechanical wounding were incubated at 15 °C and 25 °C for various times before analysis. The results showed that indole was induced quickly at 25 °C after mechanical wounding. The peak value was observed at ~4 h, with a sharp decrease thereafter. In contrast, under dual stresses, indole was induced slowly at 15 °C after mechanical wounding, reached the peak value at ~8 h, and maintained a high level for >16 h (Fig. 1B). Further, the peak indole content under dual stresses was much higher than that under single wounding stress. These results indicated that low temperature delays, but enhances, wounding-triggered indole biosynthesis.

Four homolog indole synthesis genes, namely one α-subunit homolog gene, *CsTSA*, and three β-subunit homolog genes (*CsTSB1*, *CsTSB2*, and *CsTSB3*), have been found in *C. sinensis* (Zeng *et al.*, 2016). In our previous study, a mixture of CsTSA and CsTSB2 enzymes was shown to convert indole-3-glycerol phosphate (IGP) into indole *in vitro* (Zeng *et al.*, 2016), despite the inability of either enzyme to convert IGP into indole individually. These findings suggested that *C. sinensis* indole synthase might be a protein complex. In the present study, the subcellular localization of CsTSA and CsTSB2 was investigated. CsTSA and CsTSB2 were both located in the chloroplast (Supplementary Fig. S1). This subcellular co-localization indicated that CsTSA and CsTSB2 complex formation was possible. To determine the reason for differential indole accumulation trends under dual stresses, gene expression of *CsTSA*, *CsTSB1*, *CsTSB2*, and *CsTSB3* was analyzed. The results showed that *CsTSB2* gene expression was induced by mechanical wounding. Compared with wounding stress alone, under dual stresses the expression level of *CsTSB2* was stimulated more slowly at 15 °C after wounding and was significantly higher from 4 h to 16 h (Fig. 1C). These qRT-PCR results were consistent with RNA sequencing (RNA-seq) data of tea leaves sampled after 16 h of treatment (Supplementary Fig. S2). Notably, lower indole accumulation was observed in tea leaves under dual stresses at early stages, which was consistent with the lower *CsTSB2* expression level. Therefore, weak stimulation of the *CsTSB2* expression level at early stages caused a delayed surge of indole accumulation under dual stresses. The other genes analyzed, *CsTSA*, *CsTSB1*, and *CsTSB3*, were not significantly changed. These results suggested that *CsTSB2* was the key gene, with its expression level determining the amount of indole formed. Low temperature treatment after mechanical wounding changed *CsTSB2* expression and induced a different indole accumulation pattern.

### Dual stresses enhance jasmonic acid biosynthesis

**Fig. 2.**
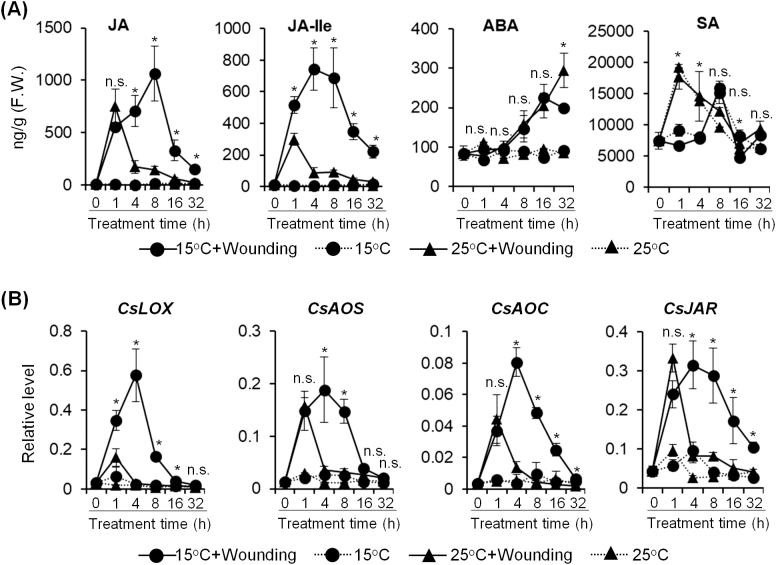
Attenuation of JA negative feedback signaling at low temperature after mechanical wounding of *C. sinensis* leaves. (A) Time course of phytohormone analysis of *C. sinensis* leaves under different treatments. Phytohormones were analyzed by UPLC-QTOF-MS. Isotope-labeled JA, ABA, and SA were used as internal standards. Asterisks (*) indicate statistically significant differences between wounded plants incubated at 15 °C and 25 °C (Student’s *t*-test; *P*≤0.05; n.s.=not significant). All data are means of three independent experiments, and error bars represent ±SD. (B) Expression level of JA biosynthesis-related genes under different treatments. The gene expression level was analyzed by qRT-PCR and calculated using the 2^−Δct^ method. * indicates statistically significant differences between a wounded plant incubated at 15 °C and 25 °C (Student’s *t*-test; *P*≤0.05; n.s.=not significant). All data are means of three independent experiments, and error bars represent ±SD.

Phytohormones are well known to be impacted by environmental stresses. However, most phytohormone responses have only been investigated under a single environmental stress. To determine the regulatory factor potentially impacting indole biosynthesis under combined wounding and temperature stress, major phytohormone content was analyzed. The results showed that, among the four major phytohormones, JA and JA-Ile levels were significantly induced by wounding, which was consistent with previous studies (Howe, 2018). Dual stresses markedly enhanced JA and JA-Ile biosynthesis, with peaks that were delayed, but significantly higher (Fig. 2A). A relatively high JA and JA-Ile level could still be detected after 32 h under dual stresses. A similar pattern to the aforementioned indole synthesis suggested a possible connection between the two responses. In contrast, ABA only responded to the mechanical damage, and no regular pattern was observed for SA (Fig. 2A). 13-Lipoxygenase (13-LOX), allene oxide synthase (AOS), allene oxide cyclase (AOC), and jasmonic acid-amido synthetase (JAR) were the key genes involved in JA and JA-Ile biosynthesis from α-linolenic acid. qRT-PCR analysis showed that these genes were all up-regulated more significantly in tea leaves under dual stresses than in those under wounding stress alone (Fig. 2B), which was in agreement with RNA-seq data generated from samples collected after 16 h of treatment (Supplementary Fig. S3). Further, dual stresses maintained the expression of these genes at a high level for longer than wounding alone. Expression changes for these biosynthesis genes were highly correlated with the trends for JA and JA-Ile content. These results suggested that JA biosynthesis was enhanced and negative feedback signaling was weakened under dual stress treatment.

### CsMYC2a regulates indole biosynthesis in jasmonic acid signaling

**Fig. 3.**
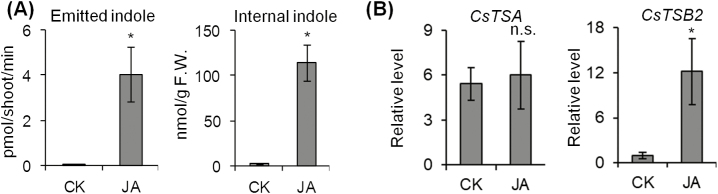
JA-induced accumulation of internal and emitted indole. (A) Indole analysis of *C. sinensis* leaves after JA treatment. Tea shoots were treated with 2.5 mM JA for 10 h. Internal indole was then extracted with CH_2_Cl_2_ and emitted indole was collected by solid-phase microextraction (SPME). Isotope-labeled indole was used as an internal standard. Asterisks (*) indicate statistically significant differences (Student’s *t*-test; *P*≤0.05). CK, control. All data are means of three independent experiments, and error bars represent ±SD. (B) Gene expression level of *CsTSB2* stimulated by JA. The gene expression level was analyzed by qRT-PCR and calculated using the 2^−Δct^ method. * indicates statistically significant differences (Student’s *t*-test; *P*≤0.05; n.s.=not significant). CK, control. All data are means of three independent experiments, and error bars represent ±SD.

Since indole and JA displayed similar dynamic change and JA has been reported to drive the synthesis of many plant volatiles (Hopke *et al.*, 1994; Rodriguez-Saona *et al.*, 2001; Ament *et al.*, 2004; Miller *et al.*, 2005), we asked if indole accumulation was induced by JA. To answer this, tea shoots were treated with 2.5 mM JA and the indole content was analyzed. The results showed that the production of both internal and emission indole was induced by exogenous JA application (Fig. 3A). The expression of *CsTSB2* also soared after JA treatment (Fig. 3B), in agreement with indole accumulation. These results showed that exogenous JA application could stimulate indole biosynthesis. Combined with increased JA and JA-Ile found in tea leaves under dual stresses, our study indicated that endogenous JA regulated indole formation under the dual stresses of low temperature and wounding.

**Fig. 4.**
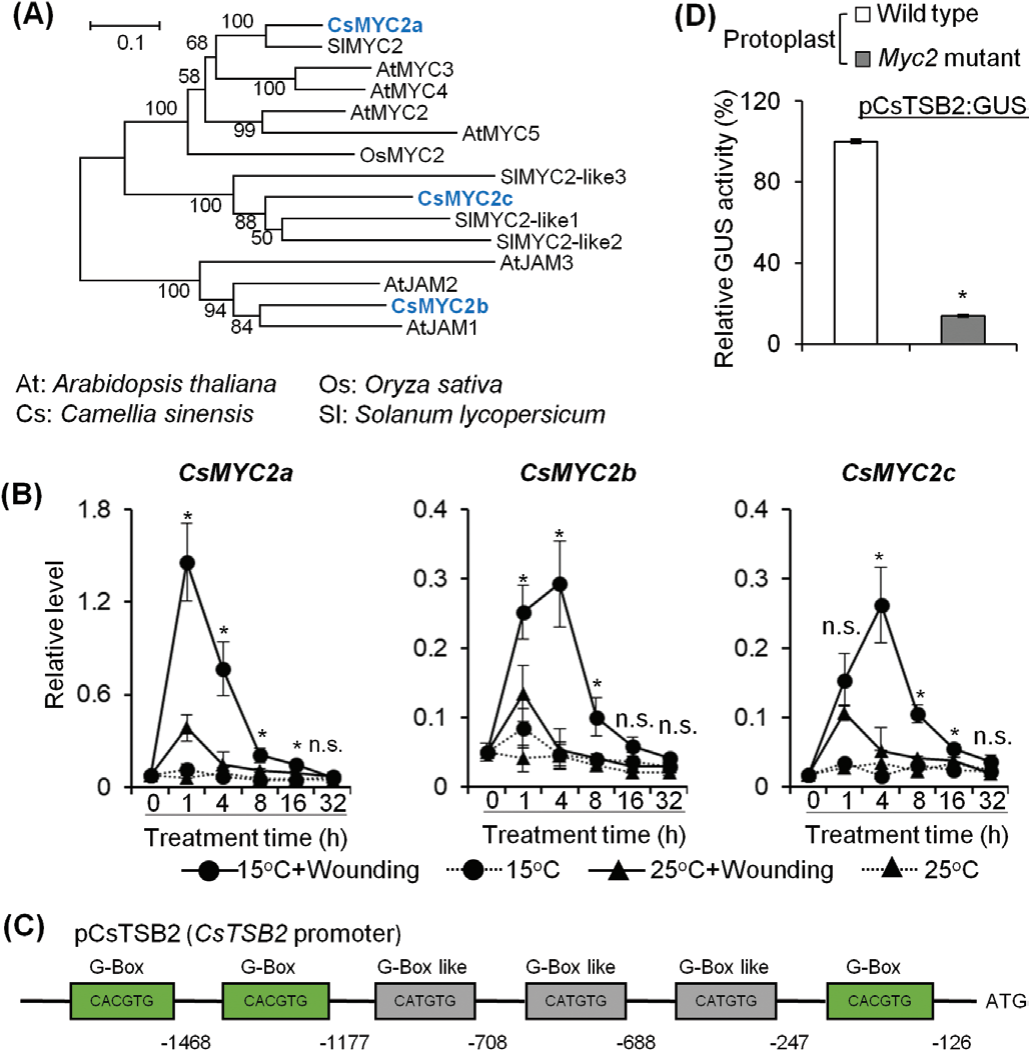
Up-regulation of homolog genes of Arabidopsis *MYC2* in *C. sinensis* under dual stresses. (A) Phylogenetic analysis MYC2 homolog protein in different plants. The Neighbor–Joining tree was constructed with ClustalW (1000 bootstrap replications). JAM, JASMONATE-ASSOCIATED MYC2-LIKE. (B) Arabidopsis *MYC2* homolog genes in *C. sinensis* were all up-regulated under dual stresses. The gene expression level was analyzed by qRT-PCR and calculated using the 2^−Δct^ method. Asterisks (*) indicate statistically significant differences between wounded plants incubated at 15 °C and 25 °C (Student’s *t*-test; *P*≤0.05). All data are means of three independent experiments, and error bars represent ±SD. (C) G-boxes and G-box-like *cis*-elements in the *CsTSB2* promoter. (D) Activation of *CsTSB2* expression by Arabidopsis MYC2. *pCsTSB2*:*GUS* was transformed into Arabidopsis wild-type Col-0 and *myc2* mutant (*jin1-8*) protoplasts. *35S:LUC* was used as an internal control. * indicates statistically significant differences (Student’s *t*-test; *P*≤0.05). All data are the means of three independent experiments, and error bars represent ±SD. (This figure is available in color at *JXB* online.)

Next, we attempted to identify the key transcription factor regulating the indole synthase gene *CsTSB2*. MYC2 is the core transcription factor in JA signaling. Three Arabidopsis *MYC2* homolog genes were found in the *C. sinensis* genome. Among these three CsMYC2s, CsMYC2a clustered with the reported functional MYC2 from Arabidopsis (Dombrecht *et al.*, 2007) (Fig. 4A). qRT-PCR analysis showed that the expression level of all three MYC2s increased dramatically under dual stresses (Fig. 4B). In addition, a MYC2-binding site, three G-boxes, and three G-box-like *cis*-elements were found in the *CsTSB2* promoter (Fig. 4C). Their presence suggested that MYC2 may mediate indole synthesis by regulating CsTSB2. Since *C. sinensis* lacks a genetic transformation system and Arabidopsis protoplast transactivation assays have been widely used to study genes from other plants, such as *Fagopyrum tataricum* and *Lotus corniculatus* (Zhou *et al.*, 2016, 2017), the Arabidopsis *myc2* (*jin1-8*) mutant (Hou *et al.*, 2010) and wild-type Col-0 were used in transactivation assays to test our hypothesis. A plasmid containing a 1854 bp *CsTSB2* promoter linked to a *GUS* gene was constructed as a reporter. Relative GUS activity showed that higher GUS activity was found in wild-type protoplasts (Fig. 4D), which indicated that *CsTSB2* transcription was regulated by Arabidopsis MYC2 protein.

**Fig. 5.**
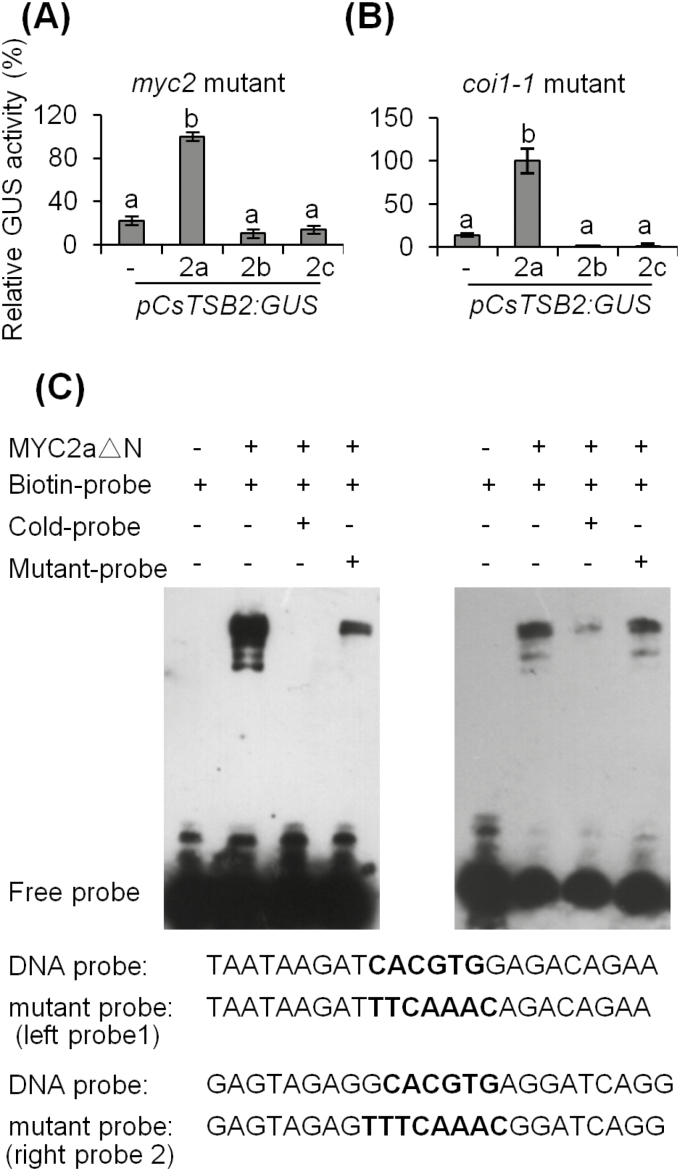
Effect of CsMYC2a on *CsTSB2* expression. (A) Transient transactivation assays showing that CsMYC2a can activate *CsTSB2* expression in the Arabidopsis *myc2* mutant mesophyll protoplast. *35S:LUC* was used as an internal control. 2a, CsMYC2a; 2b, CsMYC2b; 2c, CsMYC2c. Different letters indicate statistically significant differences (one-way ANOVA followed by Duncan’s multiple test, *P*≤0.05). All data are means of three independent experiments, and error bars represent ±SD. (B) Transient transactivation assays showing that CsMYC2a can activate *CsTSB2* expression in Arabidopsis *coi1-1* mutant mesophyll protoplast. *35S:LUC* was used as an internal control. 2a, CsMYC2a; 2b, CsMYC2b; 2c, CsMYC2c. Different letters indicate statistically significant differences (one-way ANOVA followed by Duncan’s multiple test, *P*≤0.05). All data are means of three independent experiments, and error bars represent ±SD. (C) Binding of MYC2a protein to the *CsTSB2* promoter on EMSA. Two biotin-labeled CsTSB2 promoter fragments containing the wild-type G-box were used as probes. Probe 1 is located at 1482–1459 bp upstream from the ATG initiation codon. Probe 2 is located at 1191–1168 bp from the ATG initiation codon. The unlabeled fragment (100-fold excess) or the fragment with a mutant G-box (100-fold excess) were used as competitors. −, absence; +, presence. The probe sequence is shown below the EMSA image, with the wild-type and mutant G-box in bold letters.

We then sought to determine if CsMYC2 regulated *CsTSB2* expression. *35S:CsMYC2* genes and *pCsTSB2:GUS* constructs were co-transformed into protoplasts of Arabidopsis *myc2* and *coi1-1* mutants and relative GUS activity was compared (Supplementary Fig. S6A). The results showed that CsMYC2a, but not CsMYC2b and CsMYC2c, could regulate *CsTSB2* transcription in both JA biosynthesis and signaling mutant backgrounds, indicating that CsMYC2a acts as a positive regulator of *CsTSB2* gene expression (Fig. 5A, B). To determine if the G-boxes and G-box-like *cis*-elements in the *CsTSB2* promoter are CsMYC2a-binding sites, the *cis*-elements were substituted with 5'-CGATGG-3'. GUS activity was clearly reduced in *myc2* mutant protoplasts harboring *35S:CsMYC2a* and *mpCsTSB2:GUS* (Supplementary Fig. S7), suggesting that these *cis*-elements were important for CsMYC2a binding at the *CsTSB2* promoter. To identify if MYC2a protein directly binds to the *CsTSB2* promoter, an EMSA was performed. As the full-length CsMYC2a protein was not expressed in significant quantities, we expressed and purified a truncated CsMYC2aΔN that preserved the C-terminal bHLH domain (Shoji and Hashimoto, 2011). The deleted N-terminus of CsMYC2a is essential for JAZ–MYC2 interaction, while the remaining bHLH domain is critical for MYC2 heterodimerization and binding to the G-box sequence in target promoters (Kazan and Manners, 2013). The results showed that CsMYC2aΔN formed a shifted band with the biotin-labeled DNA fragment that contained a G-box (Fig. 5C). Excess unlabeled probe eliminated the shifted band, while unlabeled mutant probe was unable to abolish the binding signal. The EMSA results suggested that CsMYC2a bound to the *CsTSB2* promoter, and the G-box was critical for this interaction.

### Role of JAZ and ICE proteins in regulating CsTSB2 under dual stress

**Fig. 6.**
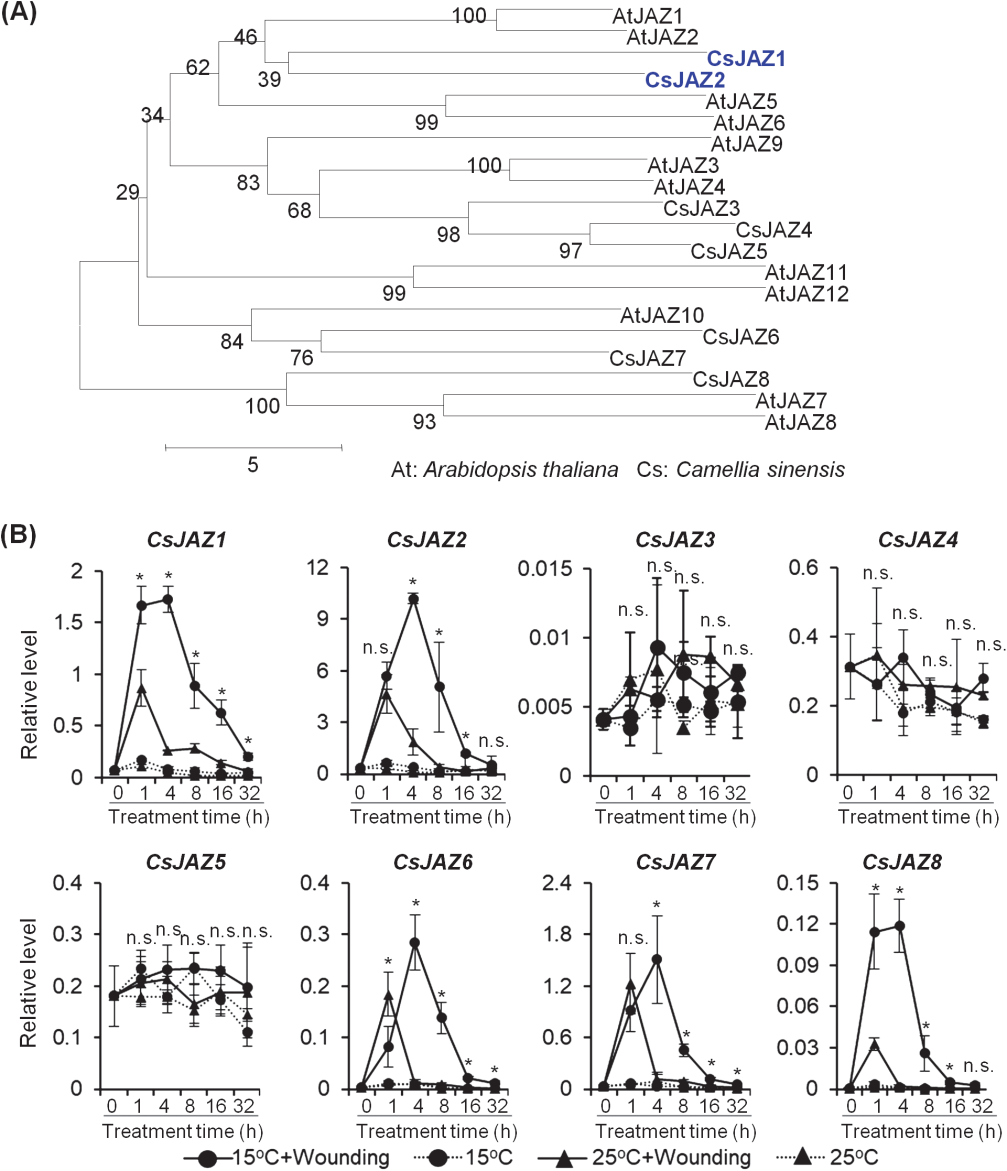
Up-regulation of *CsJAZ* gene expression under dual stresses. (A) Phylogenetic analysis of JAZ homolog protein in different plants. The Neighbor–Joining tree was constructed with ClustalW (1000 bootstrap replications). (B) Up-regulation of *JAZ* homolog genes in *C. sinensis* under dual stresses. The gene expression level was analyzed by qRT-PCR and calculated using the 2^−Δct^ method. Asterisks (*) indicate statistically significant differences between wounded plants incubated at 15 °C and 25 °C (Student’s *t*-test; *P*≤0.05; n.s.=not significant). All data are means of three independent experiments, and error bars represent ±SD.

**Fig. 7.**
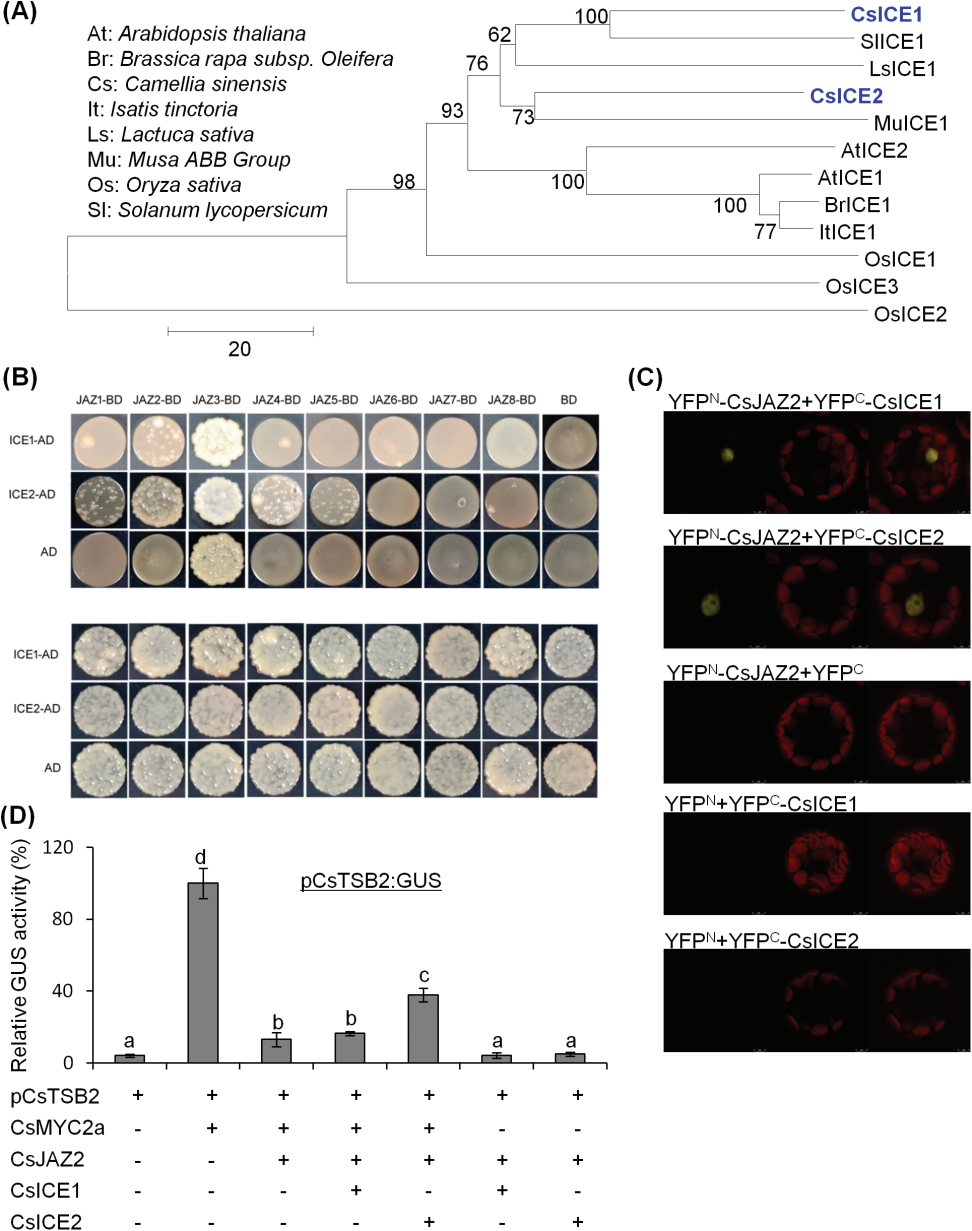
Effect of CsJAZ2 and CsICE2 interaction on CsJAZ5 inhibition of CsMYC2 and *CsTSB2* expression. (A) Phylogenetic analysis of ICE homolog protein in different plants. The Neighbor–Joining tree was constructed with ClustalW (1000 bootstrap replications). (B) Yeast two-hybrid assay showing the interaction of CsJAZs and CsICEs. BD, pGBKT7; AD, pGADT7. (C) BiFC analysis showing the interaction of CsJAZ2 and CsICEs in the Arabidopsis mesophyll protoplast. From left to right: GFP channel; chlorophyll autofluorescence; and merged images. (D) Transient transactivation assays showing that *CsTSB2* expression activation by CsMYC2a is modulated by CsJAZ2 and CsICE2 in the Arabidopsis *myc2* mutant mesophyll protoplast. *pCsTSB2:GUS* was co-transformed with other constructs into Arabidopsis *myc2* mutant mesophyll protoplasts. *35S:LUC* was used as an internal control. Different letters indicate statistically significant differences (one-way ANOVA followed by Duncan’s multiple test, *P*≤0.05). All data are means of three independent experiments, and error bars represent ±SD. (This figure is available in color at *JXB* online.)

ICE is a core regulator of cold response that mediates transcription of CBF in plants (Chinnusamy *et al.*, 2003; Zarka *et al.*, 2003). Although *ICE* is constantly expressed in low temperature, the ICE protein content can be induced by cold stress (Nakamura *et al.*, 2011). A previous study showed that ICE interacts with JAZs, the well-characterized repressors of MYC2, in Arabidopsis (Hu *et al.*, 2013). JAZ degradation induced by increased endogenous JA under cold conditions could release the ICE protein to enhance plant cold resistance by activating *CBF/DREB1* gene expression and downstream cold-responsive genes (Hu *et al.*, 2013). Here, CsJAZs and CsICEs were analyzed to determine if they played a role in indole biosynthesis under dual stresses. Eight *CsJAZ* homolog genes were identified in *C. sinensis* (Fig. 6A). qRT-PCR results showed that expression levels of most *CsJAZ* genes, except for *CsJAZ3*, *CsJAZ4*, and *CsJAZ5*, were significantly up-regulated under dual stresses (Fig. 6B). Among the eight CsJAZs, CsJAZ1 and CsJAZ2 showed the highest homology with AtJAZ1 (Fig. 6A), a key repressor of JA signaling (Chini *et al.*, 2007; Thines *et al.*, 2007). Two *ICE* homolog genes were found in *C. sinensis* (Fig. 7A). Consistent with a previous study, *CsICE* genes were constantly expressed under different stresses (Supplementary Fig. S8). As the addition of low temperature could change the dynamic expression of *CsTSB2*, the potential regulation of *CsTSB2* by CsICEs was also analyzed. *35S:CsICE* genes and *pCsTSB2:GUS* constructs were co-transformed into Arabidopsis wild-type Col-0, *ice1* mutant (Chinnusamy *et al.*, 2003), and *myc2* mutant protoplasts to compare relative GUS activities. The results showed that CsICEs had little effect on *CsTSB2* expression (Supplementary Fig. S9).

As expected, yeast two-hybrid assay results showed that most CsJAZs interacted with CsICE2. The strongest interaction was found between CsJAZ2 and CsICE2 (Fig. 7B). CsJAZ2 also interacted with CsICE1. BiFC confirmed a direct interaction between CsJAZ2 and CsICEs in the nuclei of living Arabidopsis Col-0 mesophyll protoplasts (Fig. 7C). To test the effect of CsJAZ2 and CsICEs on CsMYC2a transcriptional activation of *CsTSB2*, we performed a transient transactivation assay with the *CsTSB2* promoter (pCsTSB2) fused to a *GUS* reporter gene. Constructs for CsMYC2a, CsJAZ2, CsICE1, and CsICE2, with expression controlled by a *35S* promoter, were co-transformed with the pCsTSB2 reporter construct into Arabidopsis *myc2* mutant protoplasts (Supplementary Fig. S6B). The results showed that CsJAZ2 significantly inhibited the induction of *CsTSB2* by CsMYC2a (Fig. 7D). A yeast two-hybrid assay and BiFC experiment demonstrated an interaction between CsJAZ2 and CsMYC2a (Supplementary Fig. S10). It was remarkable that CsICE2 relieved the repression caused by CsJAZ2 (Fig. 7D). Based on these results, we concluded that the interaction of CsICE2 and CsJAZ2 released CsMYC2a to activate *CsTSB2* expression, thereby promoting indole biosynthesis under dual stresses.

## Discussion

Previous studies have shown that low temperatures and wounding alter the primary and secondary metabolic processes of plants (Kaplan *et al.*, 2004; Vladimir *et al.*, 2008; Jacobo-Velázquez *et al.*, 2015). In those studies, the quantitative association of metabolite changes and single-stress severity was investigated; however, the effect of combined application of these two different stresses on plant metabolic processes remains unclear. In response to environmental stress, plants can release blends of volatile organic compounds as a defense (Kessler and Baldwin, 2001). Our present study showed that indole, considered an insect resistance volatile in plants, responded uniquely under the dual stresses of wounding plus cold temperature, with higher indole content maintained for a longer time than under wounding alone. Increased *CsTSB2* gene expression tracked with higher indole levels in wounded leaves incubated at 15 °C. Our previous study showed that a mixture of CsTSA and CsTSB2 proteins catalyzed indole formation *in vitro* (Zeng *et al.*, 2016), suggesting that CsTSA and CsTSB2 might form a protein complex in *C. sinensis*, similar to the α _2_β _2_ tetramer formed in bacteria (Rhee *et al.*, 1996). Unlike *C. sinensis*, homologs of indole synthesis α subunit TSA were found to contribute to indole accumulation in other plants, such as *Zea mays* and Arabidopsis (Frey *et al.*, 2000; Brader *et al.*, 2001). This suggested that the indole accumulation mechanism was not conserved among plants. *CsTSB2* gene expression was significantly lower than *CsTSA* gene expression in *C. sinensis* leaves under normal growth conditions (Supplementary Fig. S11). Therefore, CsTSB2 content might be the limiting factor for CsTSA–CsTSB2 protein complex formation. A putative mechanism for CsTSB2-mediated indole formation involves increased *CsTSB2* expression boosting the level of the CsTSA–CsTSB2 protein complex, which then catalyzes indole synthesis.

JA and JA-Ile levels showed the same trend as indole content under dual stresses. JA has been reported to be one of the key phytohormones that mediate plant response to biotic and abiotic stresses (Hu *et al.*, 2013; Zhou and Memelink, 2016). Mechanical wounding, commonly caused by herbivore attack, induces JA accumulation in plants (Peña-Cortés *et al.*, 1995; Mithöfer *et al.*, 2005). Recent studies have also shown that the increased JA helps plants cope with cold stress (Du *et al.*, 2013; Hu *et al.*, 2013). Although mechanical wounding and cold both increased JA content in plants, our results did not show a significant additive effect on JA content when mechanical wounding and low temperature stress were applied together. Instead, low temperature (15 °C) was found to maintain the relatively high JA level induced by mechanical wounding for a longer time. In the absence of other stresses, rapid JA accumulation was observed after mechanical wounding, with a peak value after ~1 h (Chung *et al.*, 2008), which was confirmed in the present study. Owing to negative feedback regulation, such as that from JAZ protein (Chini *et al.*, 2007; Chung *et al.*, 2008), the JA content then rapidly decreased to initial levels. However, JA content declined more slowly in wounded leaves incubated at 15 °C. The expression of most JA and JA-Ile biosynthesis genes stayed at a higher level for longer times in leaves under dual stresses, suggesting relatively enhanced JA and JA-Ile biosynthesis. To investigate if sustained JA content at low temperature after mechanical wounding was a common phenomenon in plants, JA levels were also assayed in Arabidopsis leaves incubated at different temperatures after mechanical wounding. The results showed that a higher JA level was maintained longer when Arabidopsis plants were incubated at 4 °C after mechanical wounding (Supplementary Fig. S12). About half of the peak JA level was detected in Arabidopsis leaves after 32 h of 4 °C incubation. A lower temperature may be required to maintain JA content in Arabidopsis leaves because Arabidopsis is a relatively cold-insensitive plant (Cheng *et al.*, 2007). This result suggested that the cold temperature effect that maintains JA content after mechanical wounding might be conserved in plants. However, owing to differences in cold sensitivities, the temperature required for this effect may vary among plants.

This study confirmed that indole was regulated by JA signaling. JA treatment increased the indole content and *CsTSB2* expression level in tea leaves. Considering that JA and indole levels followed the same trend under dual stresses, dual stresses appeared to impact indole formation via JA signaling. Indole is the direct precursor of tryptophan synthesis. A previous study proposed that MYC2 negatively regulated the tryptophan synthesis pathway (Dombrecht *et al.*, 2007). However, our study showed that *CsTSB2* gene expression was positively regulated by CsMYC2a. Further, in our study, the transactive activity of CsMYC2a was rescued by CsICE2 in the presence of a CsJAZ2 inhibitor. The interaction of JAZ and ICE might explain the attenuated JA negative feedback in plants incubated at lower temperatures after mechanical wounding. ICE protein content was induced by cold stress, even when no significant change in mRNA level was observed (Chinnusamy *et al.*, 2003; Nakamura *et al.*, 2011). The ICE protein that accumulated under low temperature stress was presumed to bind with JAZ protein to attenuate the inhibitor effect of JAZ on MYC2. Then, relatively high amounts of free MYC2 stimulated JA biosynthesis, downstream MYC2-regulated gene expression, and, thereby, metabolite responses, such as volatile indole formation.

Owing to complex growth environments, plants must be capable of coping with various stresses, including simultaneous multiple stresses. A response induced by the first stress can enhance plant resistance to subsequent stress, which has been addressed (Frost *et al.*, 2008*a*, *b*). Furthermore, more robust responses are observed in plants overcoming combined stresses. For example, applying herbivore attack and drought simultaneously to *Alnus glutinosa* significantly enhanced the emission of green leaf volatiles and monoterpenes compared with either stress applied alone (Copolovici *et al.*, 2014). Volatiles were first reported as anti-insect metabolites. Further study has also identified a positive role in plant cold resistance for volatiles. (Copolovici *et al.* (2012) reported that cold stress can result in increased emission of green leaf volatiles, such as (*Z*)-3-hexenol, which is a well-characterized herbivore-induced plant volatile that plays important roles in plant–insect interactions (Farag *et al.*, 2005; Kang and Wei, 2011; Cofer *et al.*, 2018*b*). Recently, a study showed that (*Z*)-3-hexen-1-yl acetate, a subsequent metabolite of (*Z*)-3-hexenol, could promote expression of several cold stress-related genes and enhance *Z. mays* cold tolerance (Cofer *et al.*, 2018*a*). These results suggested that volatiles might play roles in resistance to multiple stresses. Therefore, as an extension of its anti-insect function, roles for indole in other environmental stress responses, such as cold resistance, need be studied.

**Fig. 8.**
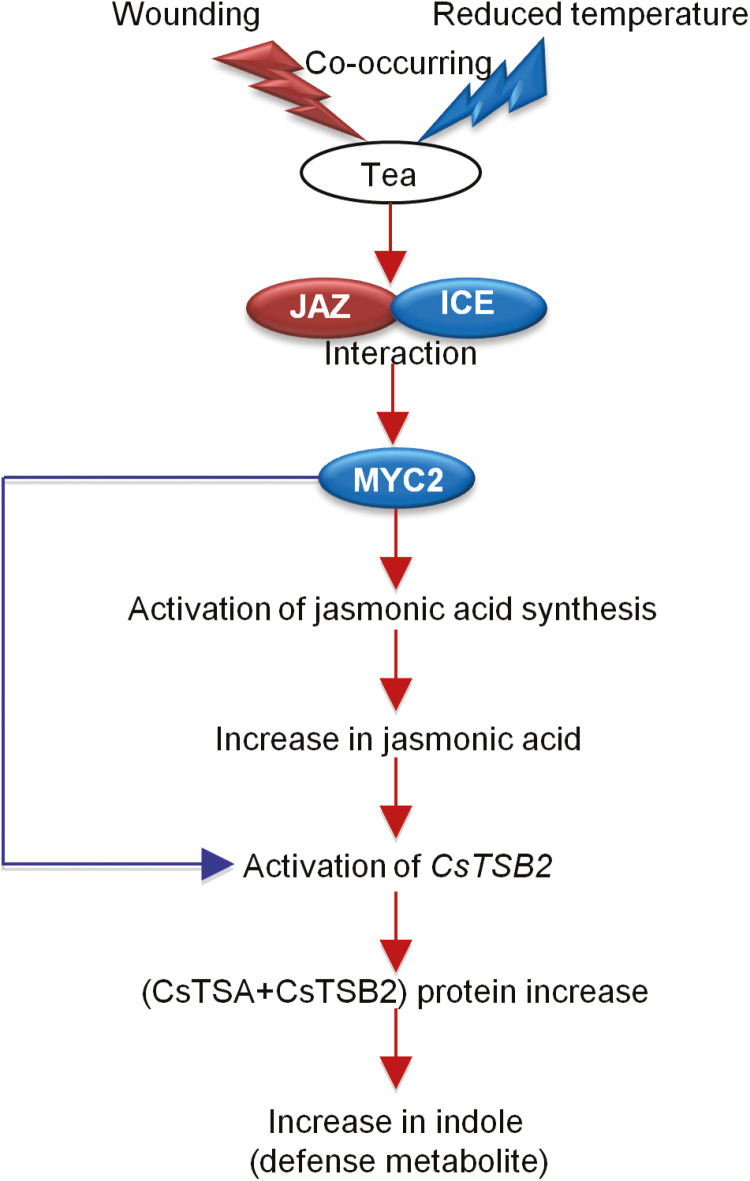
Proposed model of JA and indole formation in tea leaves exposed to dual stresses (wounding and low temperature). Under dual stresses, the competitive binding of CsICE2 to CsJAZ2 attenuates the inhibitor effect of CsJAZ2 on CsMYC2a, releasing more free CsMYC2a. CsMYC2a accelerates the expression of downstream responsive genes and promotes JA biosynthesis and indole formation. (This figure is available in color at *JXB* online.)

The present study proposed a model for JA and indole formation in tea leaves exposed to the dual stresses of wounding and low temperature (Fig. 8). Low temperature promoted the maintenance of high JA and indole levels triggered by mechanical wounding. The higher abundance of JA, the major anti-insect signaling component, and indole, an important anti-insect metabolite in plant defense, indicated a potentially stronger plant defense. Therefore, the low-temperature environment might enhance plant defense against herbivore attack. Conversely, higher JA content elicited by herbivore attack could improve the cold resistance of plants. The maintenance of JA content at low temperatures not only might help a plant to defend against herbivores, but also improve its cold resistance. These results suggested that plants may have developed an economic single defense mechanism for multiple stresses. Although the function of indole in cold resistance remains unclear, it might play an active role. Future research will focus on the effect of delayed attenuation of JA and indole on plant resistance to stress. Furthermore, analysis of the RNA-seq database generated with samples collected 16 h after various treatments indicates that, in addition to CsMYC2s, many other transcription factors were also highly up-regulated under dual stresses. Most of these transcription factors belong to the WRKY, MYB, and bHLH families. Potential roles in JA signaling have been shown for transcription factors in all three of these families (Lee *et al*., 2001; Shim *et al.*, 2012; Schluttenhofer *et al.*, 2014; Goossens *et al.*, 2017). Further study of these transcription factors might reveal additional JA-induced responses in tea leaves. Further, many other secondary metabolite synthesis-related genes were induced by dual stresses, indicating that the metabolism of stressed tea leaves changed dramatically. A combination of transcriptomics and metabonomics could be used to profile the physical changes in tea induced by dual stresses.

## Supplementary data

Supplementary data are available at *JXB* online.

Table S1. Primers for qRT-PCR used in this study.

Fig. S1. Subcelluar location of CsTSA, CsTSB1, CsTSB2, and CsTSB3.

Fig. S2. Expression level of indole biosynthesis-related genes under different treatments at 16 h from RNA-seq data.

Fig. S3. Expression level of JA biosynthesis-related genes under different treatments at 16 h from RNA-seq data.

Fig. S4. Expression level of transcription factor CsMYC2 genes under different treatments at 16 h from RNA-seq data.

Fig. S5. Expression level of *CsJAZ* genes under different treatments at 16 h from RNA-seq data.

Fig. S6. Various constructs used in transient transactivation assays.

Fig. S7. Effect of G-box mutation in the *CsTSB2* promoter on transcription activity of CsMYC2a to *CsTSB2*.

Fig. S8. Expression level of *CsICE1* and *CsICE2* under different stresses.

Fig. S9. Effect of CsICEs on *CsTSB2* expression.

Fig. S10. Interaction of CsMYC2a with CsJAZ2.

Fig. S11. Gene expression levels of *CsTSB2* and *CsTSA* in different tissues of *C. sinensis*.

Fig. S12. Dynamic change of major phytohormones in Arabidopsis leaves incubated at different temperatures after mechanical wounding.

erz570_suppl_Supplementary_Materials
