# Supplementary material for: Low temperature synergistically promotes wounding-induced indole accumulation by INDUCER OF CBF EXPRESSION-mediated alterations of jasmonic acid signaling in Camellia sinensis
Source: J Exp Bot. 2020 Jan 4;71(6):2172–85. doi: 10.1093/jxb/erz570 (PMC7242085; doi:10.1093/jxb/erz570)
Supplement: erz570_suppl_Supplementary_Materials [file erz570_suppl_supplementary_materials.pdf]

Title: Low temperature synergistically promotes wounding-induced indole accumulation by INDUCER OF CBF EXPRESSION-mediated alterations of jasmonic acid signaling in *Camellia sinensis*

Running title: Dual stresses and indole formation in tea

Authors: Ying Zhou [1, 2, †], Lanting Zeng [1, 2, †], Xingliang Hou [1], Yinyin Liao [1, 2], Ziyin Yang [1, 2, *]

***Affiliation*:**

[1] *Key Laboratory of South China Agricultural Plant Molecular Analysis and Genetic Improvement & Guangdong Provincial Key Laboratory of Applied Botany, South China Botanical Garden, Chinese Academy of Sciences, Xingke Road 723, Tianhe District, Guangzhou 510650, China*

[2] *Center of Economic Botany, Core Botanical Gardens, Chinese Academy of Sciences, Xingke Road 723, Tianhe District, Guangzhou 510650, China*

[†] These authors equally contributed to this work.

[*] **Corresponding author:** Ziyin Yang, South China Botanical Garden, Chinese Academy of Sciences, Xingke Road 723, Tianhe District, Guangzhou 510650, China; Tel: +86-20-38072989; Email address: zyyang@scbg.ac.cn.

Supplementary data

Table S1. Primers of qRT-PCR used in this study

	Gene	Primer
	CsAOC RF	GCCAATCTCAGTCATCATCATCAA
	CsAOC RR	GGAGCCACCATTAGTAACTTCAC
	CsLOX RF	AAGCAAGACTCAATCACACCAT
	CsLOX RR	GGCGTTCACCAACTCGTTA
	CsAOS RF	ACAGAGTCATACCAGAGTTCCA
	CsAOS RR	GCTTTCCCTTCACTACCCAAT
	CsJAR RF	CCACAACTCCACTCCAGAAC
	CsJAR RR	TGGCTCGTGAAGTCAACAAC
	CsJAZ1 RF	CTTCTACAGGCGTCAGTTCAA
	CsJAZ1 RR	CCCTCTCCTTGCGTTTCTC
	CsJAZ2 RF	CTGTAGTTGTTGTGAAGGAAGAGG
	CsJAZ2 RR	TGAATCCAGAAGCAGCAAGTG
	CsJAZ3 RF	AGGCTCAGGCTATTATGTTCTTG
	CsJAZ3 RR	GCGTATTAGTAGGCTGGTTGAC
	CsJAZ4 RF	CCTCAGGCTCGCTTACTCA
	CsJAZ4 RR	GCTATTGGCACTGGCAGAT
	CsJAZ5 RF	CGTCTGGTTCTGCTGATTCC
	CsJAZ5 RR	TGGTTGTCGTTGCGGTTG
	CsJAZ6 RF	GAGGAGGAATTGTAACTTGGAACT
	CsJAZ6 RR	GGTGGTGGTGGTTGTAATCG
	CsJAZ7 RF	ATCAACATCAACTTCTGGAGACTC
	CsJAZ7 RR	GGACAACCATAAGGCGACAA
	CsJAZ8 RF	TGAAGACCGTGATCGCATCT
	CsJAZ8 RR	CCGAAGGCAGGAGAATAGAGA
	CsMYC2a RF	CGCAGATATTCAACCAGGAGAG
	CsMYC2a RR	ACGGAGGATTGCCAGAAGA
	CsMYC2b RF	CCTATCAGAGCCAAGCCAATG
	CsMYC2b RR	TCTCCTCCGTCTTCCTAACAG
	CsMYC2c RF	CACCACCAACACTCCAACAA
	CsMYC2c RR	GCCGTCAAGCCAAGAGAG
	CsTSA RF	ACCACACCTACTACTCCAACA
	CsTSA RR	CTTACAGATACACGAGCACCAG
	CsTSB1 RF	TCCCGGAATGAACTGGAATA
	CsTSB1 RR	ATGTGCTCGGTTTGGCTTAG
	CsTSB2 RF	CCTTATCTCCACGCCCACTA
	CsTSB2 RR	ACGACTATGCCGACTTGAAG
	CsTSB3 RF	TCAAAGGTAAGGAGCAACAGC
	CsTSB3 RR	TAGAGCCAGCACCGTGAGAT
	CsEF RF	GTGTGGAGAAGAAGGACCCA
	CsEF RR	CGAGGCTAGTGAACAGCAAC

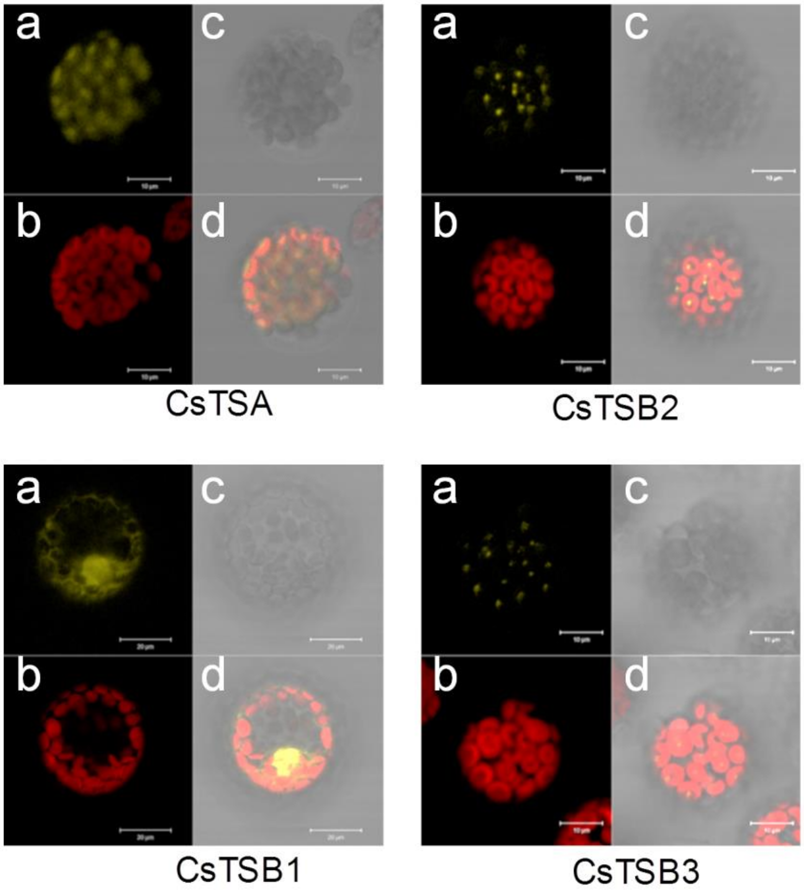

Figure S1 Subcelluar location of CsTSA, CsTSB1, CsTSB2 and CsTSB3.

The YFP-fused protein was transiently expressed in Arabidopsis mesophyll protoplasts. CsTSA, CsTSB2 and CsTSB3 were expressed in chloroplast, while CsTSB1 was expressed in cytoplasma and nucleus. a, YFP channel; b, chlorophyll auto fluorescence; c, bright field; d, merged images.

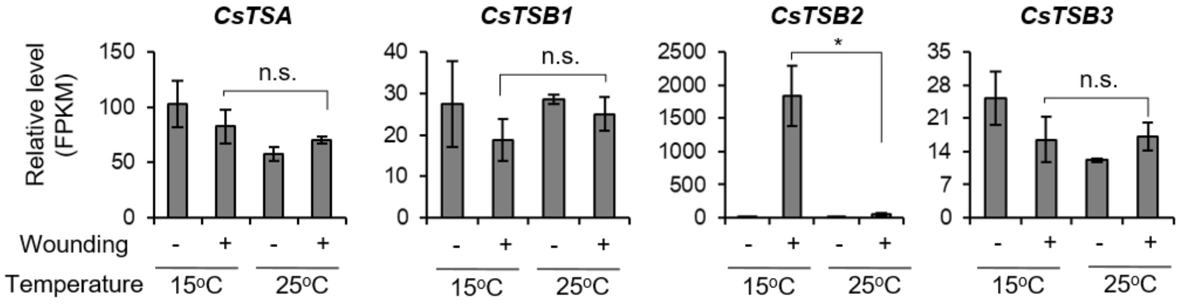

Figure S2 Expression level of indole biosynthesis-related gene under different treatments at 16 h from RNA-seq data.

The gene expression level was analyzed by transcriptome analysis. The gene expression value was expressed as FPKM (fragments per kilobase million) value. * indicates statistically significant differences (Student's *t*-test; ***p*≤0.05; n.s. = not significant). Data are means of three independent experiments and error bars represent ±SD.

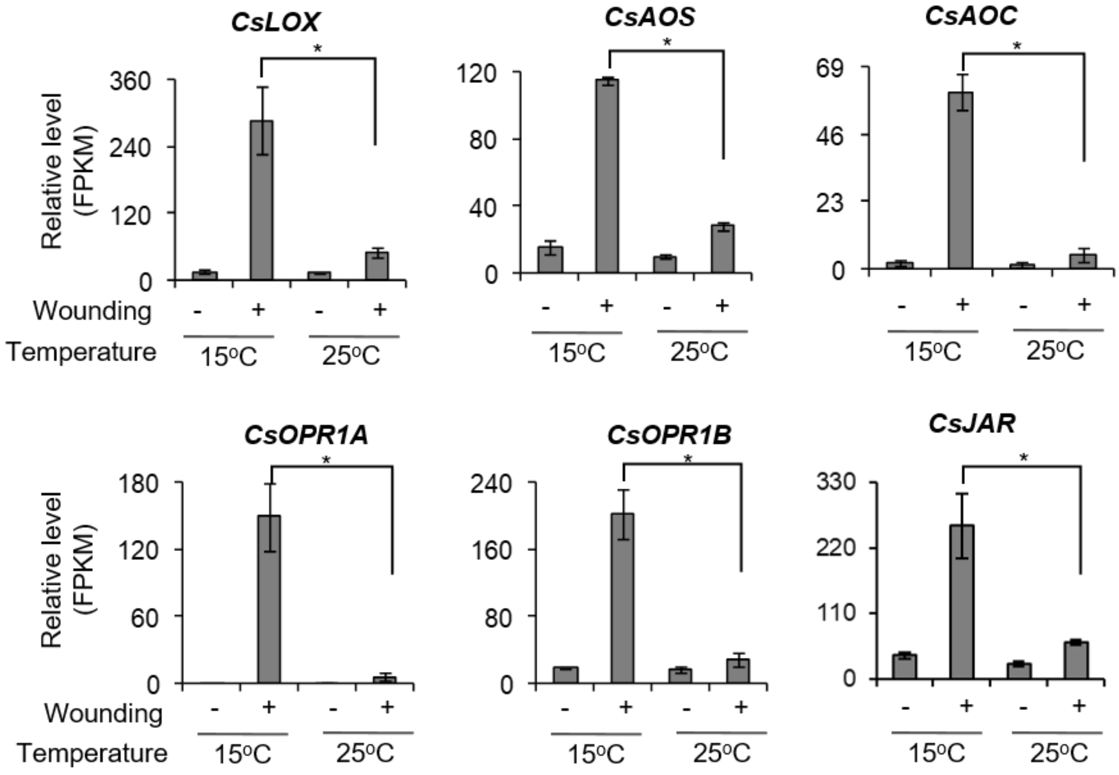

Figure S3 Expression level of JA biosynthesis-related gene under different treatments at 16 h from RNA-seq data.

The gene expression level was analyzed by transcriptome analysis. The gene expression value was expressed as FPKM (fragments per kilobase million) value. CsLOX, 13-lipoxygenase; CsAOS, allene oxide synthase; CsAOC, allene oxide cyclase; CsOPR, 12-oxophytodienoate reductase; CsJAR, jasmonic acid-amido synthetase. * indicates statistically significant differences (Student's *t*-test; **$p \leq 0.05$). Data are means of three independent experiments and error bars represent ±SD.

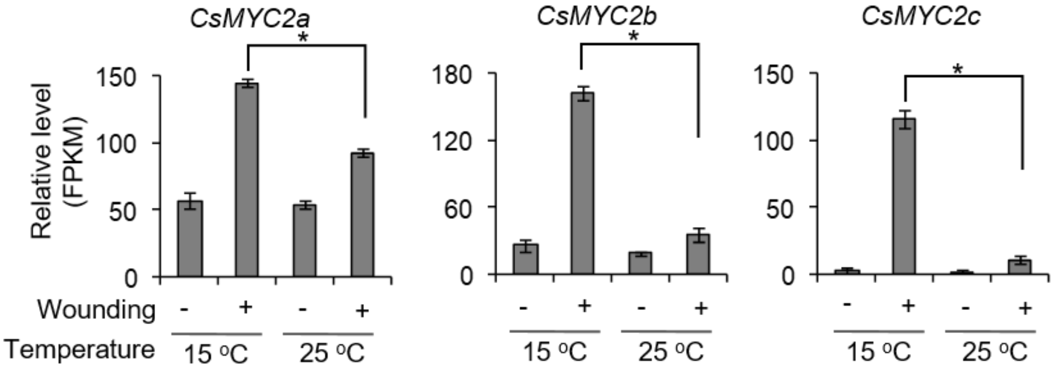

Figure S4 Expression level of transcription factor *CsMYC2s* gene under different treatments at 16 h from RNA-seq data.

The gene expression level was analyzed by transcriptome analysis. The gene expression value was expressed as FPKM (fragments per kilobase million) value. * indicates statistically significant differences (Student's *t*-test; ***p*≤0.05). Data are means of three independent experiments and error bars represent ±SD.

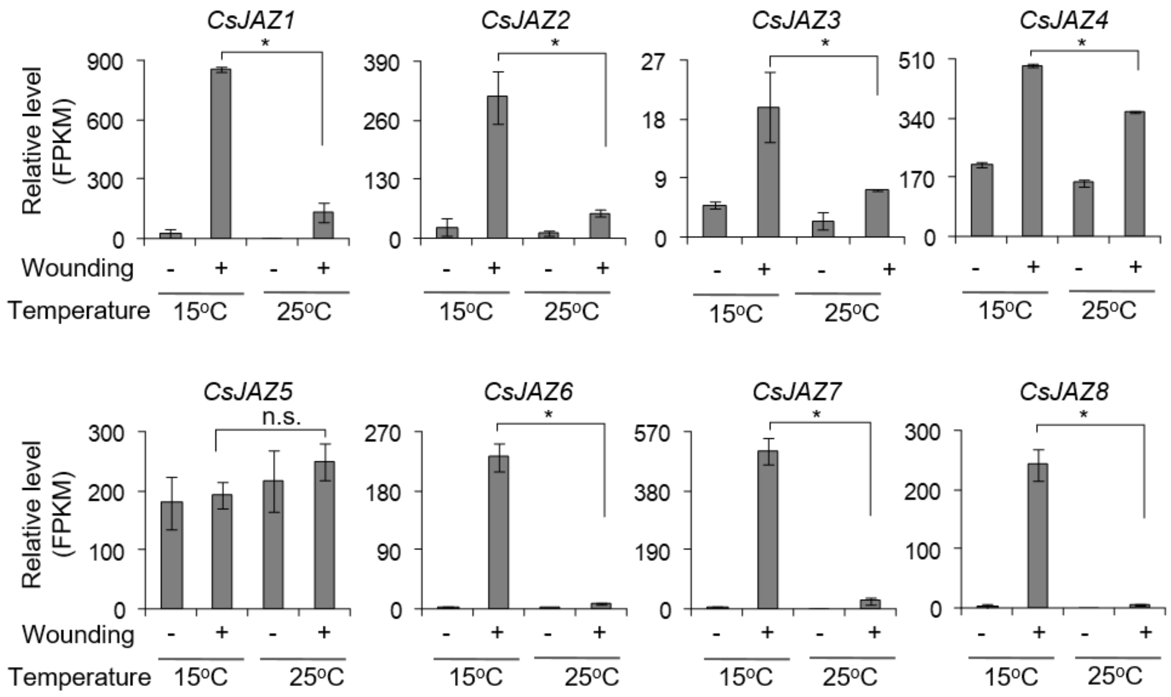

Figure S5 Expression level of *CsJAZs* gene under different treatments at 16 h from RNA-seq data. The gene expression level was analyzed by transcriptome analysis. The gene expression value was expressed as FPKM (fragments per kilobase million) value. * indicates statistically significant differences (Student's *t*-test; **$p \leq 0.05$; n.s. = not significant). Data are means of three independent experiments and error bars represent ±SD.

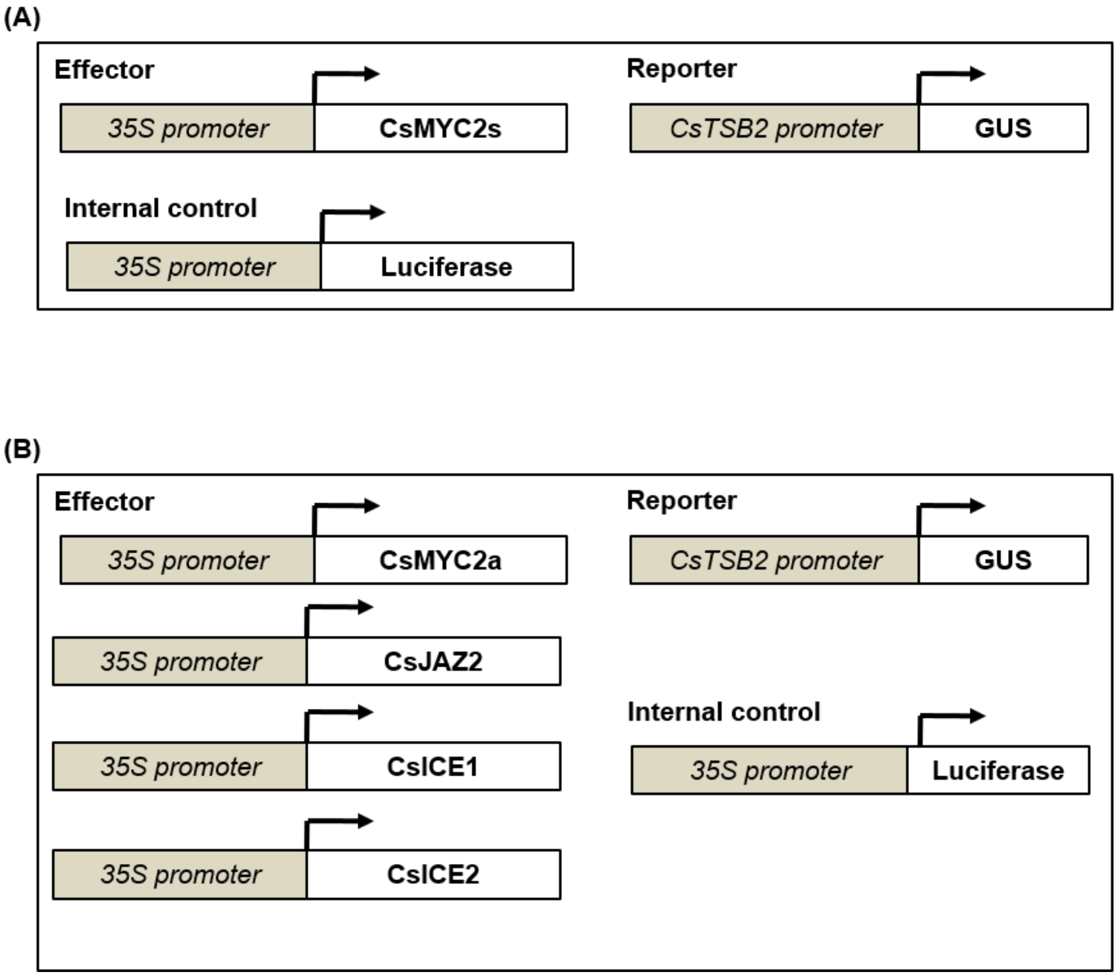

Figure S6 Various constructs used in transient transactivation assays.

(A) Constructs used in Figure 5a and 5b. (b) Constructs used in Figure 7d.

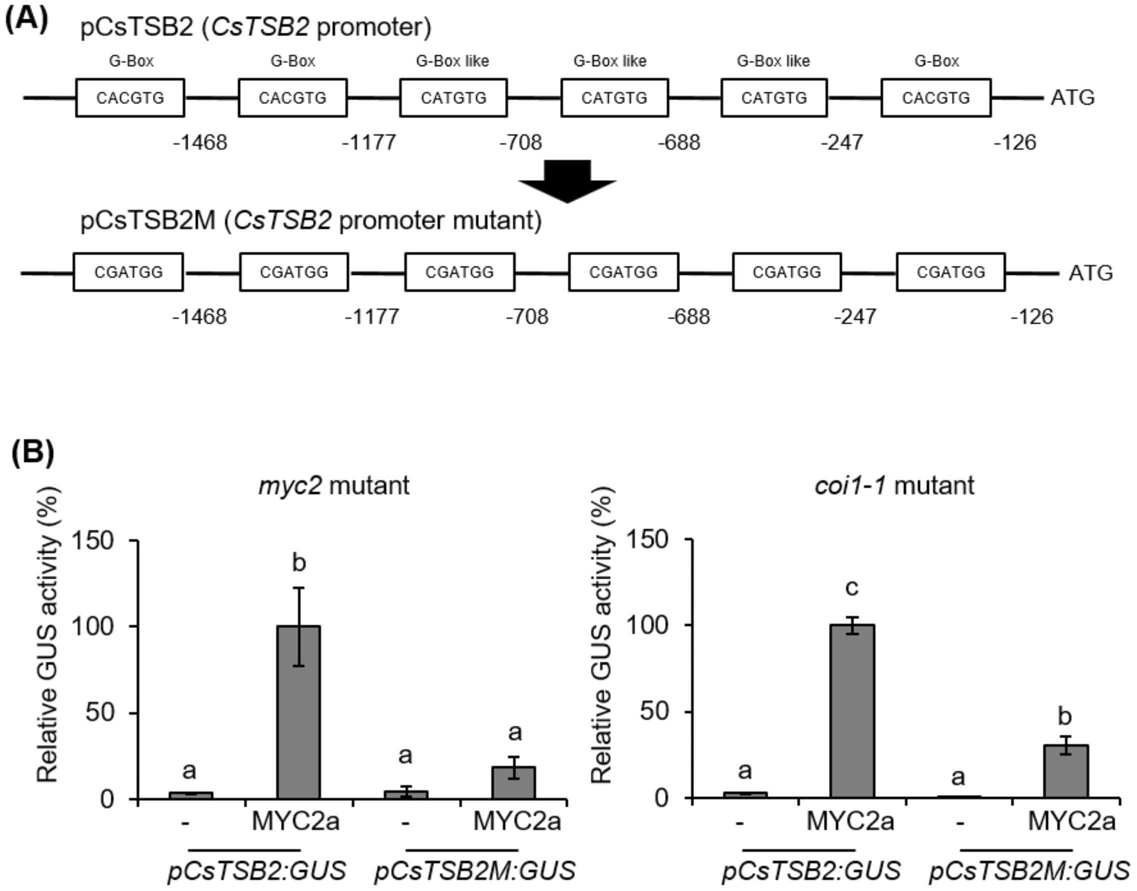

Figure S7 Effect of G-box mutation in *CsTSB2* promoter on transcription activity of CsMYC2a to *CsTSB2*.

(A) Mutation of G-boxes and G-box-like elements in *CsTSB2* promoter. (B) Transient transactivation assays show that CsMYC2a lost binding activity to the mutated *CsTSB2* promoter significantly, and significantly reduced transcription activity of CsMYC2a to *CsTSB2* was observed both in *Arabidopsis myc2* and the *coi1-1* mutant mesophyll protoplast. *35S:LUC* was used as internal control. Different letters indicate statistically significant differences (one-way ANOVA followed by Duncan' multiple test, $p \leq 0.05$). All data are means of three independent experiments and error bars represent ±SD.

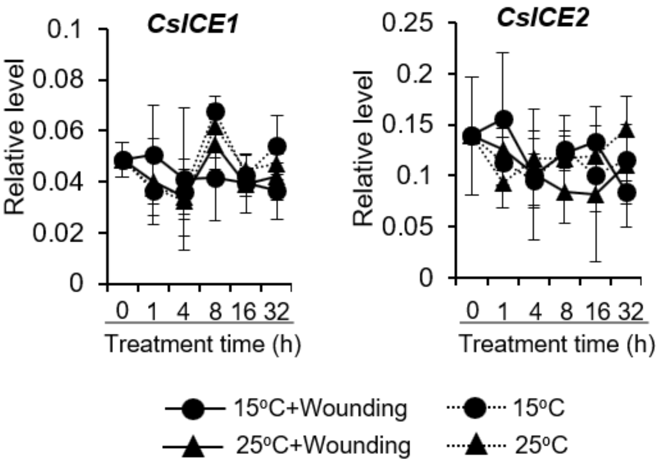

Figure S8 Expression levels of *CsICE1* and *CsICE2* under different stresses.

The gene expression level was analyzed by qRT-PCR and calculated using the $2^{-\Delta ct}$ method. All data are means of three independent experiments and error bars represent ±SD.

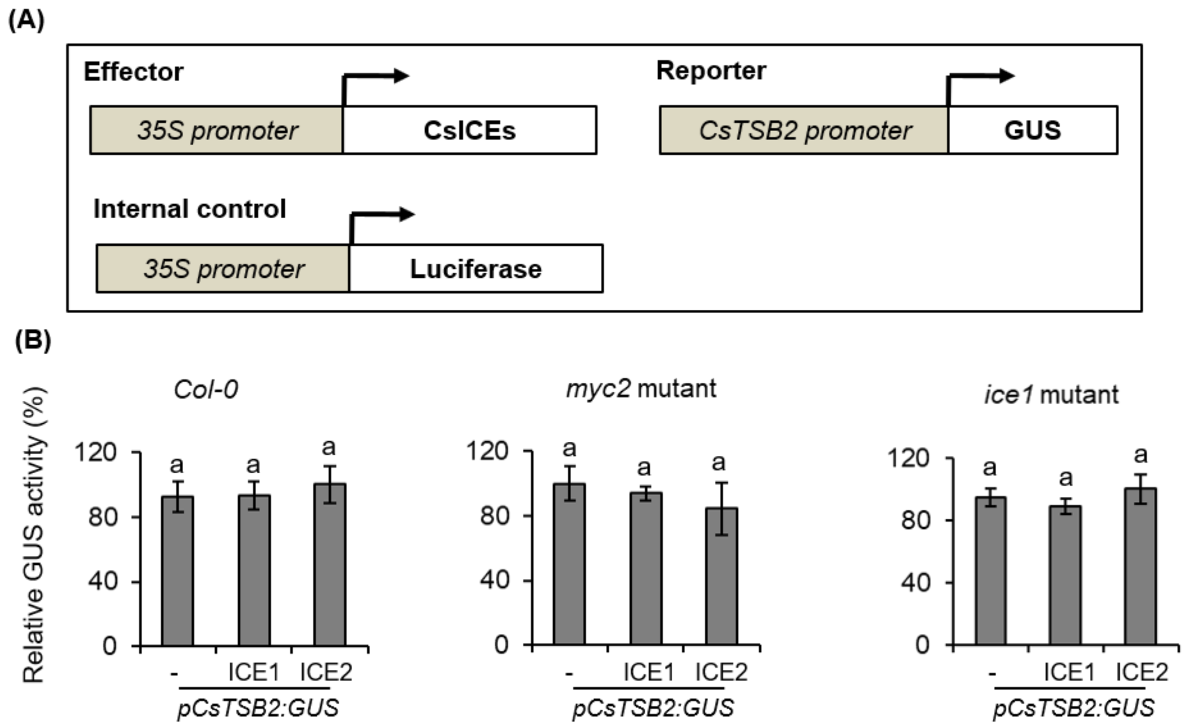

Figure S9 Effect of CsICEs on *CsTSB2* expression.

(A) Constructs used in Figure S8B. (B) Transient transactivation assays show that CsICEs could not activate *CsTSB2* expression in *Arabidopsis Col-0, myc2* mutant and *coi1-1* mutant mesophyll protoplast. *35S:LUC* was used as internal control. Different letters indicate statistically significant differences (one-way ANOVA followed by Duncan' multiple test, $p \leq 0.05$). All data are means of three independent experiments and error bars represent ±SD.

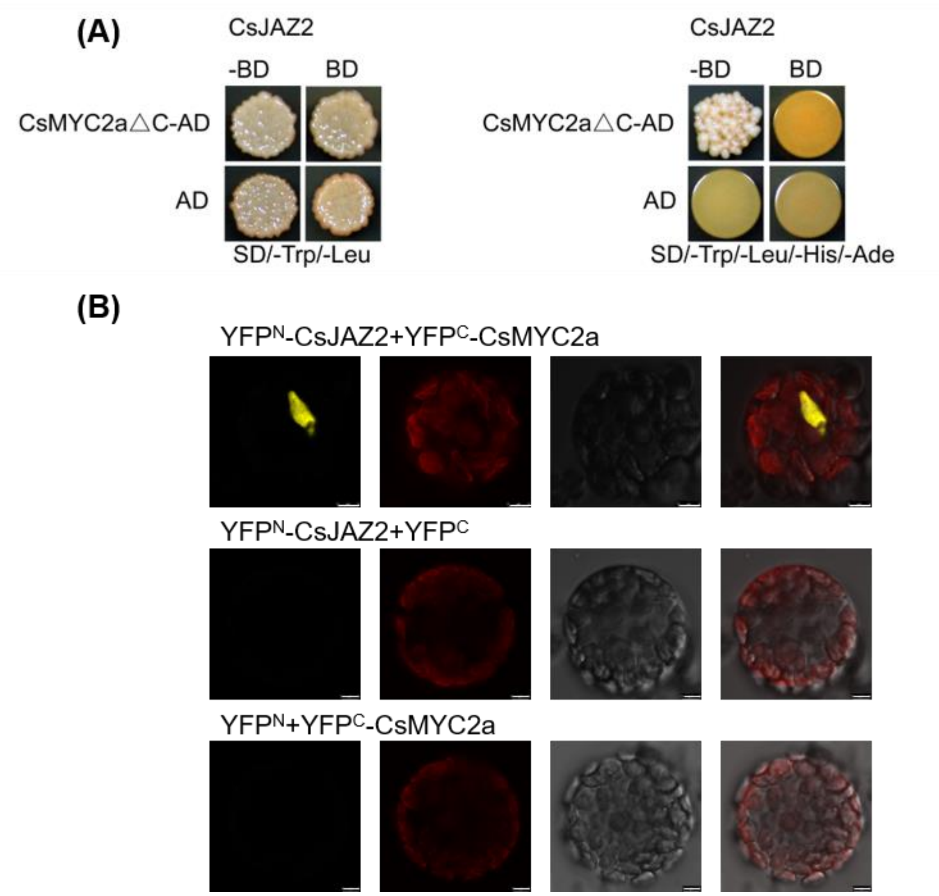

Figure S10 Interaction of CsMYC2a with CsJAZ2.

(A) Yeast two-hybrid assay showed the interaction of CsMYC2a and CsJAZ2. Because the full-length CsMYC2a protein exhibited strong self-activation activity, the truncated version of CsMYC2a protein (corresponding to amino acid residues 1-200), which abolished CsMYC2a self-activation completely, was chosen. BD, pGBKT7; AD, pGADT7. (B) BiFC analysis showed the interaction of CsMYC2a and CsJAZ2 in the *Arabidopsis* mesophyll protoplast. From left to right: YFP channel, chlorophyll autofluorescence, bright field, merged images. Scale bar, 5 μm.

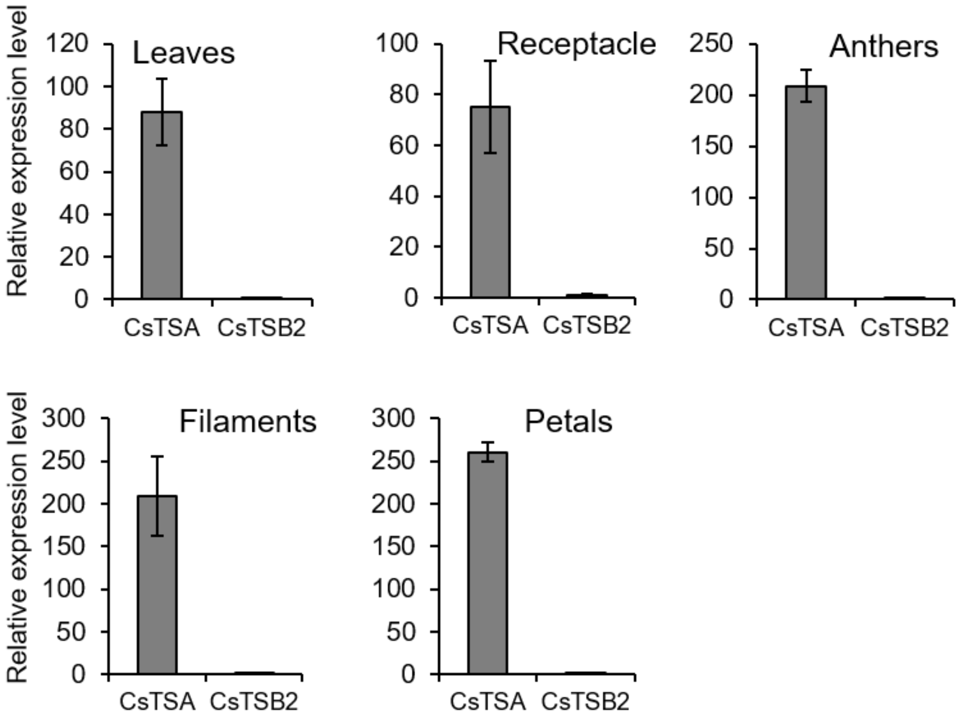

Figure S11 Gene expression levels of *CsTSB2* and *CsTSA* in different tissues of *C. sinensis*. Tea flower was dissected into receptacle, filament, petal and anther. Gene expression level was analyzed by qRT-PCR. $2^{-\triangle\triangle ct}$ method was used to analyze the data.

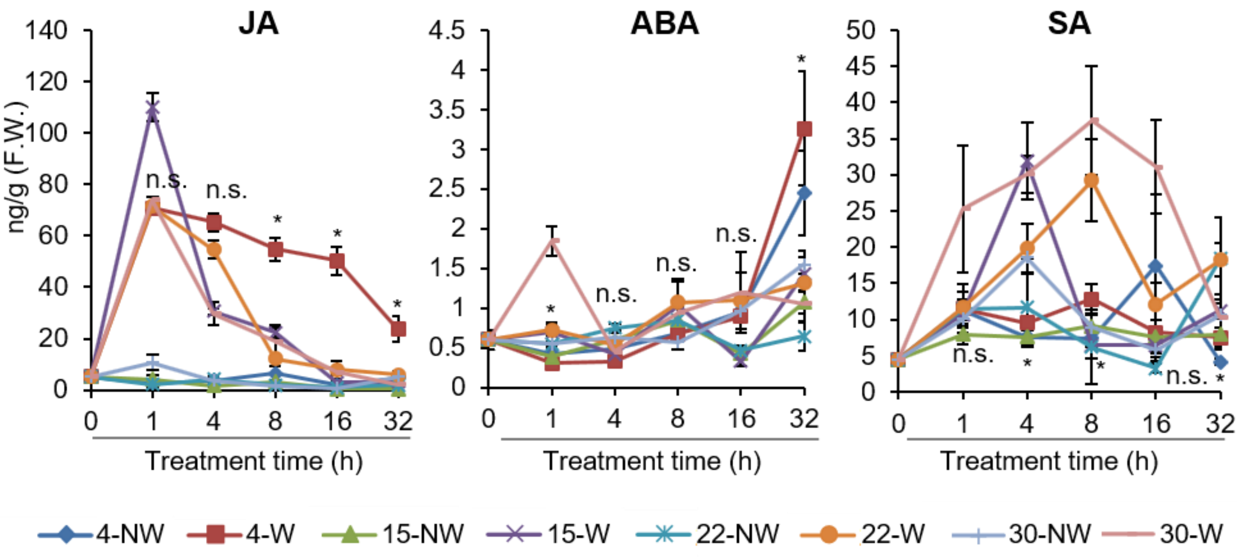

Figure S12 Dynamic change of major phytohormones in *Arabidopsis* leaves incubated at different temperature after mechanical wounding.

Arabidopsis leaves were wounded using a syringe needle. The *Arabidopsis* plants were then incubated at different temperatures, including 4, 15, 22, and 30 °C after mechanical wounding. *Arabidopsis* plants incubated at 22 °C served as control. Leaves were collected at 0, 1, 4, 8, 16, and 32 h. The phytohormones of leaves, including JA, SA, and ABA, were analyzed by UPLC-QTOF-MS. Isotope-labeled phytohormone was used as internal standard. * indicates statistically significant differences between wounded plants incubated at 4 and 22 °C (Student's *t*-test; * $p \leq 0.05$; n. s. = not significant). All data are means of three independent experiments and error bars represent ±SD. NW: not wounded; W: wounded.
